# Effect of central serous chorioretinopathy patients by covering sick eye for 48 hours

**DOI:** 10.5935/0004-2749.20220011

**Published:** 2025-08-21

**Authors:** Hongwei Zhao, Changyu Qiu, Yuanyuan Shi, Yan Zhang, Chuang Nie, Yubo Gong, Jun Zhao, Ling Luo

**Affiliations:** 1 Department of Ophthalmology, The 306th Hospital of People’s Liberation Army, Beijing, China; 2 Department of Ophthalmology, The Seventh Medical Center of Chinese People’s Liberation Army General Hospital, China

**Keywords:** Central serous chorioretinopathy/therapy, Visual acuity, Coriorretinopatia serosa central/terapia, Acuidade visual

## Abstract

**Purpose:**

To assess the effect of continuously covering the sick eye affected with central serous chorioretinopathy for 48 h.

**Methods:**

This retrospective, case-control study involved 32 central serous chorioretinopathy patients categorized in the treatment group composed of 17 sick eye that received continuous covering treatment for 48 h with a medical gauze and the observation group composed of 15 of these patients who were followed up. None of the patients received any other treatments or medications. The logarithm of the minimal angle of resolution (logMAR) best-corrected visual acuity, macular retinal thickness, and the root mean square value of the amplitude density in the first ring of multifocal electroretinogram were examined before and after the 48-h treatment.

**Results:**

After the continuous treatment, the logMAR best-corrected visual acuities were 0.31 ± 0.18 and 0.56 ± 0.37 in the treatment and observation groups, respectively (p=0.019). The macular retinal thicknesses were 461 ± 43 µm and 498 ± 50 µm in the treatment and observation groups, respectively (p=0.032). The root mean square values of the amplitude density in the first ring of multifocal electroretinogram were 32.5 ± 5.3 nV/deg^2^ and 26.6 ± 4.3 nV/deg^2^ in the treatment and observation groups, respectively (p=0.002).

**Conclusions:**

The continuous application of the covering treatment for 48 h on the sick eye showed positive outcomes with respect to the best-corrected visual acuity, macular retinal thickness, and macular retina functions in the treatment of central serous chorioretinopathy.

## Abstract (pt)

**Objetivo:**

Este estudo teve como objetivo avaliar o efeito da cobertura contínua do olho doente de pacientes com coriorretinopatia serosa central por 48 horas.

**Métodos:**

Este estudo retrospectivo, caso-controle, incluiu 32 pacientes com coriorretinopatia serosa central, dos quais 17 receberam tratamento de cobertura contínua por 48 horas no olho doente com gaze médica como grupo de tratamento e 15 foram acompanhados como grupo de observação. Todos os pacientes não receberam nenhum outro tratamento ou medicamento. O logaritmo do ângulo mínimo de resolução da acuidade visual melhor corrigida (LogMar), a espessura macular da retina e o valor médio da raiz quadrada da densidade da amplitude no primeiro anel do eletroretinograma multifocal foram examinados antes e após o tratamento por 48 horas, respectivamente.

**Resultados:**

Após o tratamento contínuo, a acuidade visual melhor corrigida pela escala logMar foi de 0, 31 ± 0, 18 no grupo de tratamento e 0, 56 ± 0, 37 no grupo de observação (p=0, 019). A espessura macular da retina foi de 461 ± 43 µm no grupo de tratamento e 498 ± 50 µm no grupo de observação (p=0,032). O valor médio da raiz quadrada da densidade de amplitude no primeiro anel do eletroretinograma multifocal foi de 32,5 ± 5,3 nV/deg^2^ no grupo com cobertura e foi de 26,6 ± 4,3 NV/deg^2^ no grupo de observação (p=0,002).

**Conclusões:**

O tratamento de cobertura contínua no olho doente, durante 48 horas, apresentou efeitos positivos na acuidade visual melhor corrigida, na espessura e na função macular da retina no tratamento da coriorretinopatia serosa central.

## INTRODUCTION

Central serous chorioretinopathy (CSC) is a posterior segment disease characterized by localized and limited serous detachments of the neurosensory retina, which is often associated with focal detachments of an altered retinal pigment epithelium (RPE). In most cases, CSC is self-limited and the visual prognosis is good. However, some cases develop progressive visual loss due to persistent serous retinal detachment, cystoid macular degeneration, or RPE decomposition^(1)^.

The pathogenesis of CSC is still not fully understood. Currently, choroidal vascular hyper permeability is considered to be the major attributable reason for the increased tissue hydrostatic pressure beneath the RPE, which eventually leads to disintegration of the RPE continuity^(2)^. Hence, strengthening the barrier function of RPE may be a potential means for treating CSC.

The RPE is the main site of the visual cycle that produces a phototoxic product, especially under light stimulation during the daytime, which increases the burden on the RPE. Therefore, when the barrier function of the RPE is destructed, RPE may become disqualified for the visual cycle due to light stimulation in the daytime; thereby, even a single episode of acute CSC may result in an acute decrease in the functioning of retinal photoreceptor cells and in chronic degenerative effects on photoreceptor neurons owing to the phototoxic mechanisms, finally leading to multiple recurrences of CSC or chronic CSC with persistent fluid accumulation^(3)^. We therefore hypothesized that reducing the burden of the visual cycle on the RPE by avoiding light stimulation may yield positive outcomes in the treatment of CSC. In this study, we investigated the effect of continuous covering the sick eye for 48 h of CSC patients.

## METHODS

All experiments involving humans were performed in accordance with the ethical code and the recommendations issued by the Ethics Committee of Human Experimentation and the Helsinki Declaration of 1975 (revised in 2010).

The present study was a retrospective, case-control analysis wherein all patients underwent clinical examinations for the determinations of best-corrected visual acuity measurement (BCVA) and intraocular pressure and the slit lamp and fundus examination, fundus fluorescein angiography/indocyanine green angiography (FFA/ICGA), macular optical coherence tomography (OCT), and multifocal electroretinogram (mERG) examination. The CSC diagnosis was established using the FFA with inkblot or smokestack leakage, ICGA with hyper permeability of choroidal vasculature, and OCT with definite subretinal fluid. The patients were enrolled with acute CSC (within 3 months of onset) and were of age 30-50 years. Only those patients who had experienced only one episode of CSC and had not received any systemic and local treatments were included. The patients who did not undergo re-examination for BCVA, OCT, and mERG after 48 h of the initial examinations and those with chronic or recurrent CSC were excluded from the study.

The observation group CSC patients did not receive any treatment or medication. The treatment group CSC patients only received a single 48 h continuous treatment wherein the sick eye was continuously covered with eight layers of medical gauze. After the intervention or the observation for 48 h, the patients were re-examined for BCVA and by OCT and mERG.

The macular retinal thickness (MRT) was measured manually using calipers with the highest detachment on OCT. If neurosensory and RPE detachments overlapped on OCT, the heights of both the detachments were summed.

Data were expressed as the mean ± standard deviation. Statistical analyses were performed using a commercially available statistical software package (SPSS version 19.0). Mann-Whitney *U*-test and Fisher’s exact test were used for analyzing the nonparametric data. Statistical significance was set at p<0.05, two-tailed.

## RESULTS

A total of 32 eyes (from 32 patients) were enrolled consecutively from March 2013 to January 2017. The mean age of the patients was 41.9 ± 9.8 years. The subjects included 22 men and 10 women. The treatment group included 17 patients (17 eyes) and the observation group included 15 patients (15 eyes). The demographic data on sex, age, and the duration of current CSC episode were not significantly different between the groups. In addition, no significant differences were noted in the baseline BCVA, MRT, and root mean square (RMS) values of mERG between the groups. The baseline characteristics of both the groups are shown in table 1.

**Table 1:** Baseline characteristics of the treatment and observation groups

Characteristics	Covering	Observation p-value	
Age (yr)			0.697^*^
Gender (male:female)	12:5	10:5	0.302∆
Duration of CSC episode (Day)	36.9 ± 7.9	34.8 ± 6.5	0.422^*^
Baseline logMAR BCVA	0.62 ± 0.47	0.61 ± 0.45	0.952^*^
Baseline MRT (µm)	538 ± 149	491 ± 107	0.320^*^
Baseline RMS of the amplitude density in the first mERG ring (nV/deg^2^)	22.5 ± 3.8	23.4 ± 4.8	0.559^*^

Values are presented as mean ± SD. RMS= root mean square; SD= standard deviation; logMAR BCVA = logarithm of minimal angle of resolution best-corrected visual acuity.

* Mann-Whitney *U*-test;

∆ Fisher’s exact test.

**Figure 1:**
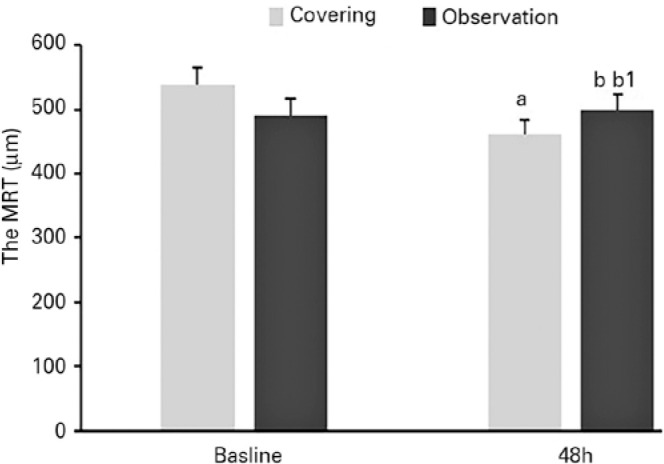
a. Compared with the baseline values, there is a statistical diffe rence in MRT after continuous covering for 48 h on the sick eye in the treatment group (p=0.048, Mann-Whitney U-test). b1. Compared with the baseline values, no statistical difference can be seen in MRT after 48 h in the observation group. (p=0.952, Mann-Whitney U-test,). b1: There exists a statistical difference in MRT between the treatment and observation groups (p=0.032, Mann-Whitney U-test).

**Figure 2:**
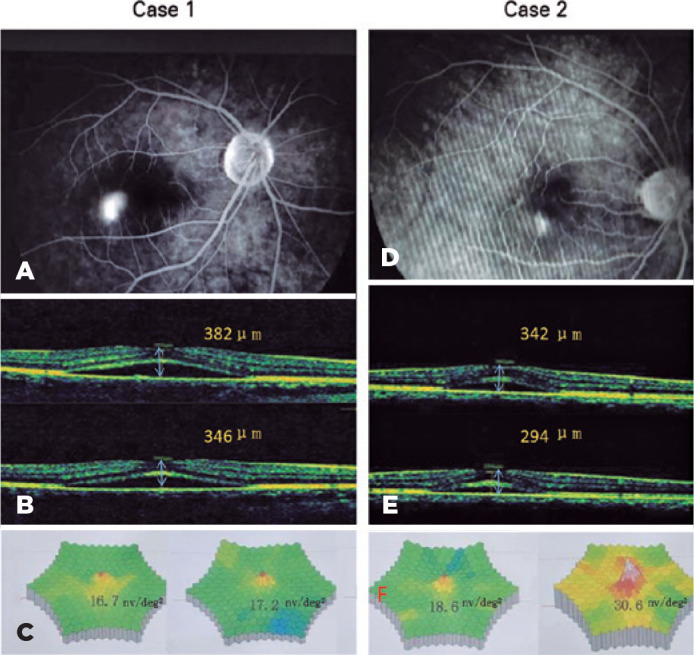
Two representative cases in the treatment group. Case 1: male sex, age of 27 years, complain of darken vision for the past 10 days. Case 2: female sex, age of 42 years, complain of decreased vision for 1 week. FFA shows the leakage in the macula before covering (A,D). OCT showing the MRT changes before (top panel) and after (lower panel) covering (B,E). mERG displaying the changes before (left panel) and after covering (right panel) in each picture (C,F).

After the intervention or observation for 48 h, the MRT in the treatment group was found to be thinner than that in the observation group (461 ± 43 µm vs 498 ± 50 µm, respectively; p=0.032, Mann-Whitney U-test). The results of MRT analysis are depicted in figure 1, and two typical cases are illustrated in figure 2.

**Figure 3:**
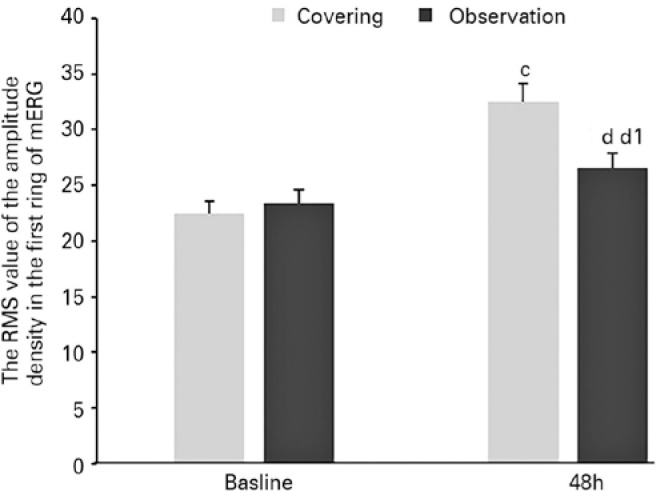
c. Compared with the baseline values, a statistical difference can be seen in the root mean square (RMS) value of the amplitude density in the first ring of mERG after continuous covering for 48 h on the sick eye in the treatment group (p=0.000, Mann-Whitney U-test). d. Compared with the baseline values, no statistical difference can be seen for the RMS value of the amplitude density in the first ring of mERG after 48 h in the observation group (p=0.065, Mann-Whitney U-test). d1. There was a statistical difference in the RMS value of the amplitude density in the first ring of mERG between the treatment and observation groups (p=0.001, Mann-Whitney U-test).

After observation for 48 h, the RMS value of the amplitude density in the first ring of mERG was found to be higher in the treatment group than in the observation group (32.5 ± 5.3 nV/deg^2^ vs. 26.6 ± 4.3 nV/deg^2^, respectively, p=0.002, Mann-Whitney U-test). The RMS values of the amplitude density in the first ring of mERG are given in figure 3, and the typical cases of the RMS value of the amplitude density in the first ring of mERG are depicted in figure 2.

**Figure 4:**
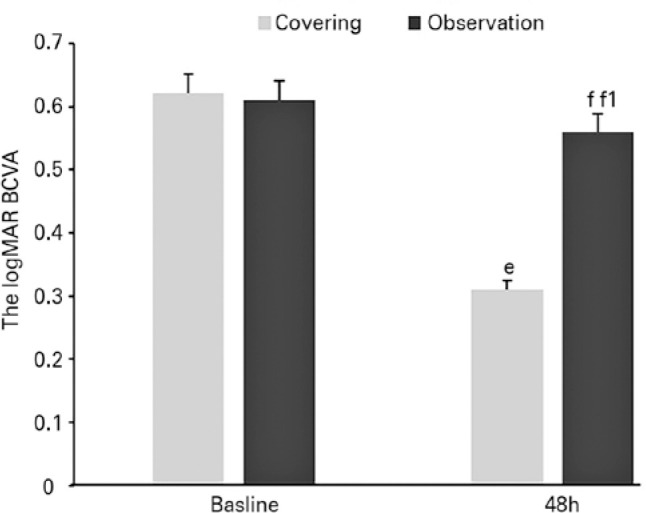
e. Compared with the baseline values, a statistical difference was noted in the logMAR BCVA value after continuous covering for 48 h on the sick eye in the treatment group (p=0.016, Mann-Whitney U-test). f. Compared with the baseline value, no statistical difference was noted in the logMAR BCVA value after 48 h of observation (p=0.074, Mann-Whitney U-test,). f1. There was a statistical difference in the logMAR BCVA between the treatment and observation groups (p=0.019, Mann-Whitney U-test).

After observation for 48 h, the logMAR BCVA value was better in the treatment group than in the observation group (0.31 ± 0.18 vs. 0.56 ± 0.37, respectively, p=0.019, Mann-Whitney U-test). The results of BCVA analysis are shown in figure 4.

## DISCUSSION

In this study, our results revealed that the BCVA value increased, the degree of macular retinal detachment decreased, and the macular retina functions improved after the continuous covering treatment of the sick eye for 48 h relative to those in the observation group. Thus, based on these results, we believe that the continuous covering of the sick eye may shorten the course of CSC, making it a potential treatment option in acute CSC patients. However, similar studies with longer follow-up duration are warranted to infer about the efficacy of the continuous covering treatment approach.

CSC occurs in five forms: acute, non-resolving, recurrent, chronic, and inactive^(4)^. Acute CSC is a self-limiting di sease, and several researchers believe that the symptoms for acute CSC last for <3 months, during which time, re-attachment of the neurosensory retina occurs in most cases^(5-7)^. Observation alone is the typical management approach for acute CSC, because patients usually experience spontaneous improvement of symptoms over a period of time. Hence, although treatment modalities such as laser photocoagulation, intravitreal injection of anti-vascular endothelial growth factor agents, and photodynamic therapy can speed up the healing process, these are usually not recommended as the first choice by ophthalmologist in the first-attack period due to economic considerations or the potential complications. Most researchers therefore affirm that observation may be the preferred approach for managing acute CSC^(8)^. However, in acute cases, subretinal fluid collection may reappear in 30%-50% of cases within 12 months of the first attack and then resolve again spontaneously^(3)^. Hence, acute CSC patients often desire a quicker and more effective resolution of their disease to avoid progression to chronic CSC, and, ultimately, facing permanent visual impairment.

Most CSC patients are men who have decreased and/or distorted vision together with color appreciation. The pathogenesis of CSC remains unclear until date. CSC is a chorioretinal disease that causes idiopathic retinal serous detachment including one or more areas of leakage from the choroid through a defect from the outer blood-retina barrier of RPE^(9,10)^. Therefore, it has been declared that the RPE and the choroid are the central contributors to subretinal fluid accumulation with subsequent detachment of the neurosensory retina^(11)^. Multiple mechanisms of focal RPE barrier breakdown are therefore likely involved in the CSC progression, such as mechanical stress resulting from increased intra-choroidal pressure or dilated choroidal vessels, reduced RPE adhesion, alteration of hydro-ionic RPE regulation, and RPE atrophy secondary to chorio capillaris hypoperfusion^(2,11,12)^, which together connotes a sustained elevation of the hydrostatic pressure present underneath the detached RPE. Hence, the RPE plays a seemingly important role in regulating the integrity of the barrier function. When the RPE barrier function cannot be repaired quickly, recurrent CSC or chronic CSC may develop. Consequently, a simple observation does not seem the best treatment approach for acute CSC patients.

RPE is the main place for the visual cycle that requires the cooperation of photoreceptor cells and RPE^(13)^. RPE damage due to the separation of neurosensory retina from the RPE in CSC affects the normal visual cycle process, produces phototoxic stress, and causes an acute decrease in the functioning of retinal photoreceptor cells as well as induces chronic degenerative effects on photoreceptor neurons owing to tehe phototoxic mechanisms^(14,15)^. The intervention of the visual cycle can suppress the phototoxic stress placed on cells involved in the visual cycle via inhibition of the key visual cycle enzymes so as to avoid the accumulation of cytotoxic compounds^(16-18)^. The above listed points form an important theoretical basis of our study and also a reasonable explanation of the study outcome.

Visual cycle intervention can be pharmacological or non-pharmacological in nature. Pharmacological intervention of the visual cycle has been developed for the treatment of various retinal disorders. These modulators can produce a substantial inhibition of the visual cycle in the body and reduce the formation of retinal toxic substances^(19)^. Some pharmacotherapy for modulating the visual cycle has been applied in the treatment of retinal diseases such as Leber congenital amaurosis, Stargardt macular dystrophy, and nonexudative age-related macular degeneration based on pre-clinical and some early clinical studies^(20,21)^. Despite the lack of reports supporting the application of this visual cycle intervention in CSC, we believe that it may be a future option for the treatment of CSC.

Another visual cycle intervention method is a non-drug intervention that blocks the physiological cycle of the visual cycle. A past study reported that the short-time suppression of the visual cycle via patching can improve the visual function, as assessed by mERG in some CSC patients. The author interpreted the results by providing the repair time of the RPE and reducing the light-induced retinal photoreceptor neuron injury^(22)^. However, the study included only eight cases, with no parallel control.

Our approach was a non-drug intervention. We found good clinical results supporting that short-term suppression of the visual cycle via continuous covering of the sick eye can reduce the MRT, which represent the degree of separation of the RPE, and improve the values of BCVA and mERG, which represent the function of macular retina in patients with CSC.

In our study, an interesting phenomenon of an obvious improvement was observed in CSC patients with a broader degree of RPE detachment via continuously covering the sick eye with respect to the values of BCVA, MRT, and mERG. This phenomenon seems to indicate that larger the fields of RPE detachment, larger is the macular retina area of the effective recovery after continuously covering the sick eye in patients with CSC. Moreover, we noted clear improvement via continuous covering of the sick eye in the shorter duration of CSC episode than that in the longer duration of the CSC episode. This situation indicated that the degree of recovery for CSC patients depends on the severity of the disease, which is closely associated with the duration of the disease.

There are some limitations in this study. The thorough disappearance of subretinal fluid that restores the normal anatomy and photoreceptor functions is commonly accepted as the principal endpoint of CSC treatment^(23)^. Consequently, our study required further follow-up to analyze whether longer continuous covering on the affected eye in CSC patients will produce better outcomes. We believe that the examinations should be performed at the same time during the day because a variation in the subretinal fluid level in CSC may affect the outcome and lead to bias. In addition, we were also unaware of the prognosis of the two groups of patients, because there was no long-term follow-up in our study. Furthermore, only acute CSC was investigated. Chronic or recurrent CSC was excluded. In fact, chronic CSC can be a terrible disease that can lead to legal blindness, which is different from that by acute CSC. Approximately 13% of chronic cases progress to legal blindness and develop a BCVA of 20/200 or worse after 10 years^(24)^. Therefore, it is possible that, with a larger study population, subgroups may be identified that reveal differing responses to visual cycle suppression via continuous covering.

In summary, our results thus demonstrated that, in acute CSC cases, continuous covering of the sick eye for 48 h can show positive outcomes in terms of the values of BCVA, MRT, and macular retina function. Further investigations are warranted to facilitate the understanding of the effectiveness of this therapy in patients with CSC.
